# A national audit of hand injuries in UK

**DOI:** 10.1016/j.jpra.2026.09.022

**Published:** 2026-09-28

**Authors:** Konstantinos Devetzis, Jennifer C E Lane, Ella Darveniza, Sara Sousi, Grey Giddins, Maxim D Horwitz, Hand Injury Prevention Collaborative, Will Mason, Alexander Curtis, Binta Jalloh, Rajesh Gopireddy, Nitin Sawant, Kudamaduwage Gomes, Jemma Rooker, Langhit Kurar, Juliana Hughes, Sayhow Teoh, Ryan Trickett, Rhodri Gwyn, Kath Hamlin, Michael David, Haneen Abed, Zaf Naqui, Christiana Lipede, Mo Akhavani, Francis Banhidy, Catrin Wigley, P Caine, Sheeva Rabie, Gursharan Khera, Jamil Moledina, Amir Labib, Alice Gargan, Maximillian Greenfield, Alistair Hunter, John Hardman, Meg Birks, Matt Weston, Dominic Power, Abdus Burahee, Adrian Murphy, Helen Wohlgemut, Simon Wimsey, George Matheron, Joshua Ong, Robert Poulter, Segun Ayeko, Sami Hassan, Khaled M. Yaghmour, David Warwick, Sherif Fetouh, Onur Berber, Rory Cuthbert, George Wheble, Emily West, Edmund Wright, Lisa Leonard, Emma Reay, Rebecca Martin, David Clarke, Sian Sokota, Ahmed Zaghloul

**Affiliations:** a Imperial College London, Faculty Building, Exhibition Road South Kensington, London, SW7 2AZ, United Kingdom; b Bone & Joint Health from Barts Bone and Joint Health, Blizard Institute, Queen Mary University of London, 4 Newark Street, E1 2AT, UK; c Nuffield Department of Orthopaedics, Rheumatology and Musculoskeletal Sciences, Nuffield Orthopaedic Centre, University of Oxford, Windmill Road, Oxford, OX3 7LD, UK; d Bayview Surgery, Avalon, NSW, Australia; e Royal United Hospital, Combe Park, Bath, BA1 3NG, UK; f Chelsea and Westminster Hospital, 369 Fulham Road, SW10 9NH, London, UK

**Keywords:** Audit, Hand, Injury, National, Prevention

## Abstract

**Introduction:**

Hand injuries are a frequent cause of morbidity, with patterns influenced heavily by societal behaviour. This audit investigated hand injuries in the UK occurring after COVID-19 restrictions were lifted (early 2022) and compared them with data from the COVID-19 lockdown period (early 2021).

**Methods:**

A multicentre cross-sectional study was conducted across 23 hospitals, with clinicians prospectively recording consecutive hand injuries over a 2-week period (February–March 2022). Eligible cases included referrals to Orthopaedic, Plastic, or Hand surgery services. Data were submitted using a standardised online proforma, and preventability was classified using predefined criteria.

**Results:**

2,309 injuries were recorded from 23 hospitals – a 21% rise. Males made up 60% of cases. Median age was 34, similar to the lockdown period of 40. Sports-related injuries increased (10.2% to 22.7%), while DIY-related injuries declined (8% to 4%). Phalangeal and metacarpal fractures were the most common injuries. Alcohol and drug-related injuries rose from 2.4% to 4.5%. Overall, preventable injuries decreased from 50% to 35%, with only 19% of injuries in children under 16 deemed preventable. Most cases (63%) were managed non-operatively, and 43% required follow-up.

**Conclusion:**

Following the lifting of COVID-19 restrictions, hand injuries increased, with a shift toward sports-related mechanisms and fewer preventable injuries. Despite this reduction, preventable injuries remain a substantial public health concern, highlighting opportunities for targeted safety interventions.

## Introduction

Hand injuries are frequent and can result in significant, long-term disabilities if not managed properly. The incidence of hand injuries in the United Kingdom (UK) has been reported to be up to 110 per 100,000 in the adult population.^1^ To date, literature has focused on the prevention of hand injuries in specific sub-groups of the population, including children.^2^^,^^3^ Some authors studied specific injuries, such as those caused by power tools and sports-related injuries.^4^, ^5^, ^6^ Whilst others collected data from a single centre in the UK.^7^ Lane et al. examined hand injuries on a national scale in the UK, demonstrated the feasibility of recording such data and provided a definition for preventability.^8^ However, the data collection took place during the COVID-19 pandemic. During lockdown, hand injury referrals saw a 68% reduction, with fewer sports-related injuries observed but a notable increase in falls, crush injuries, and injuries from recreational cycling.^9^ This unusual shift in injury patterns suggests that the dataset was influenced by the unique circumstances of this period, making it less representative of typical trends. Overall, there is a lack of comprehensive understanding of the prevalence and definition of preventable injuries on a national level. Therefore, it is of interest to study the injuries sustained following the lifting of COVID-19 restrictions to identify the epidemiology of injuries sustained outside of extenuating circumstances posed by a global pandemic.

### Aims and objectives

This audit aimed to identify the frequency of preventable hand injuries in the UK, looking at the mechanism and type of injuries sustained. The secondary aim of the project was to compare the prevalence of hand injuries in comparison to those seen during the COVID-19 lockdown. The audit also aimed to identify potential areas where injuries could be prevented to reduce the burden of injury.

## Patients and methods

### Data collection

Consultant surgeons specialising in Orthopaedics, Plastics, or Hand surgery across the UK were recruited to participate in an evaluation of preventable hand injuries over the period between 22/02/2022 and 08/03/2022. Data were collected from hospitals across 24 locations. These included District General Hospitals, Major Trauma Centres (MTC) and specialist academic and teaching institutions. These were largely the same centres included in the previous study by Lane et al. (2023). Unfortunately, the Manchester data set was excluded from the present study because the number of cases submitted was substantially lower than expected for an MTC and was therefore considered unlikely to be representative of the centre's usual case volume. To avoid introducing potential bias into the analyses, these data were excluded. Each hospital recorded the injuries of all consecutive patients presenting to their department over the 2-week period. The inclusion and exclusion criteria were maintained from the previous study.^8^ Patients with a hand injury referred to the Orthopaedic, Plastic or Hand Surgical team were included. Superficial hand lacerations with no deeper injury identified or a soft tissue injury not requiring a referral for surgical review were excluded from the data collection. The recruited clinicians completed a standardised online proforma during or at the end of each clinic.

### Ethical approval

As this project was conducted as a national clinical audit, it was classified as an audit/service evaluation using the UK Health Research Authority (HRA) Decision Tool and did not require Research Ethics Committee approval. Local registration and approval were obtained at each participating centre in line with local governance guidelines.

### Sample characteristics and definition of preventability

Data collected included patient demographics (sex, age, hand dominance), type and mechanism of injury, time of presentation from injury to attendance of emergency department (ED), time of presentation to the referral services, and management. Preventable injury criteria were decided *a priori* by the authors. A preventable hand injury was established *a priori* as ‘an injury to that hand (e.g. laceration, abrasion) that is innate to the activity being performed and that would not have occurred if reasonable human interventions were in place’. This was based on the definition given by Silber et al.^7^ Injuries occurring within the home environment—such as those related to Do-It-Yourself (DIY) activities, cooking, handling windows and doors, mechanical falls, and manual labour—were classified as preventable. This categorisation was based on the premise that such injuries occur as a result of modifiable risk factors, inadequate safety measures, or lapses in attention, and may therefore be reduced through targeted public health interventions and education. In contrast, sports-related injuries, as well as those resulting from violence or self-harm, were considered non-preventable within the scope of this study, as they are more closely associated with behavioural, psychological, or external factors that are less amenable to short-term prevention strategies. These data were entered into a spreadsheet with drop-down menus to optimise accuracy as well as a descriptor column in the case of doubt. The descriptors of the injury mechanisms were assessed to ascertain whether they were preventable or not preventable; if in doubt they were noted to not be preventable.

### Data analysis

Descriptive statistics were used to summarise patient characteristics and outcomes. Categorical variables (including mechanism of injury and management type) were compared using Pearson’s Chi-squared test. Continuous variables (including time intervals from injury to GP/ED or from presentation to specialty review) were compared using the Wilcoxon rank-sum test due to non-normal distribution. A p-value of <0.05 was considered statistically significant. All analyses compared outcomes between MTC and non-MTC groups.

## Results

Data were collected from 23 hospitals across the UK, ensuring a balanced geographical distribution and access to specialised care services (as seen in Tables 1, Table 2, Table 3). 2,309 patients satisfied the requirements of the inclusion criteria. There were 1,396 males (60.5%), and 905 females (39.2%); in eight cases (0.3%) the sex was not recorded. There were slightly more injuries on the right side (1204 versus 1,072 left-sided injuries) with bilateral injuries in 21 patients. The median age of all patients was 34 (Interquartile range (IQR) 21-55) years; the median age of the adults (≥ 16 years) was 40 (IQR 26-57) years; the median age of the children was 12 (IQR 8-13) years. The median time from injury to ED or general practice (GP) attendance was 0 days (same day) (IQR 0-2 days). The median time from attendance to ED or GP to review by a specialist was one day (IQR 0-4 days). Most of the patients were right-handed (71.3%), with the rest being left-handed (7.5%) or ambidextrous (0.7%); in 20.5% of cases, data on patients’ handedness were missing.

**Table 1:** Number of hand injuries in Major Trauma Centres (MTC).

Hospital trust	Number of hand injuries	Percentage of all hand injuries (%) (n=2309)
St George’s University Hospitals NHS Foundation Trust	211	9.1
University Hospitals Birmingham NHS Foundation Trust	158	6.8
University Hospital Southampton NHS Foundation Trust	144	6.2
Sheffield Teaching Hospitals NHS Foundation Trust	122	5.3
Cardiff and Vale University Health Board	117	5.0
Leeds Teaching Hospitals NHS Trust	105	4.5
South Tees Hospitals NHS Foundation Trust	100	4.3
University Hospitals Plymouth NHS Trust	91	3.9
University Hospitals Coventry and Warwickshire NHS Trust	85	3.7
University Hospitals Bristol and Weston NHS Foundation Trust	80	3.5
Brighton and Sussex University Hospitals NHS Trust	79	3.4
NHS Greater Glasgow & Clyde	47	2.0
Total	**1,339**	**58**

**Table 2:** Number of hand injuries in Non-Major Trauma Centres (Non-MTC).

Hospital trust	Number of hand injuries	Percentage of all hand injuries (%) (n=2309)
Chelsea and Westminster Hospital NHS Foundation Trust	200	8.6
Gloucestershire Hospitals NHS Foundation Trust	189	8.1
Royal Free London NHS Foundation Trust	146	6.3
Royal United Hospitals Bath NHS Foundation Trust	122	5.3
Western Sussex NHS Trust	79	3.4
Whittington Health NHS Trust	69	3.0
Princess Alexandra Hospital NHS Trust	47	2.0
Northampton General Hospital NHS Trust	42	1.8
University College London Hospitals NHS Foundation Trust	33	1.4
Royal Cornwall Hospitals NHS Trust	22	1.0
Great Western Hospitals NHS Foundation Trust	13	0.6
Total	**962**	**41.7**

**Table 3:** Missing data/Unknown Hospitals.

Category	Number of hand injuries	Percentage of all hand injuries (%) (n=2309)
Missing/Unspecified	8	0.4

The most frequently recorded injuries included single fractures of phalanges or metacarpals, infection, and wrist fractures (see graph 1). Graph 2 represents the mechanisms of injury, highlighting the distinction between preventable and non-preventable injuries. 798 (34.6%) of hand injuries were deemed preventable. Sports injuries (18.2%) and mechanical falls outdoors (13.9%) (both considered not preventable) were the commonest causes of injury, while in 17 patients (0.7%) there was no recorded mechanism of injury. There was a limited association of injuries with drug or alcohol use, with only 94 (4.1%) cases reported as associated with alcohol and 12 (0.5%) associated with drug use. In children, defined as <16 years of age and making up only 14% of the cohort of patients, the most common mechanism of injury was sports and the prevalence was 38.2%, followed by mechanical fall outdoors (9.9%) and door/window injury (9%). Overall, only 18.6% of injuries in children were deemed preventable.

**Fig. 1:**
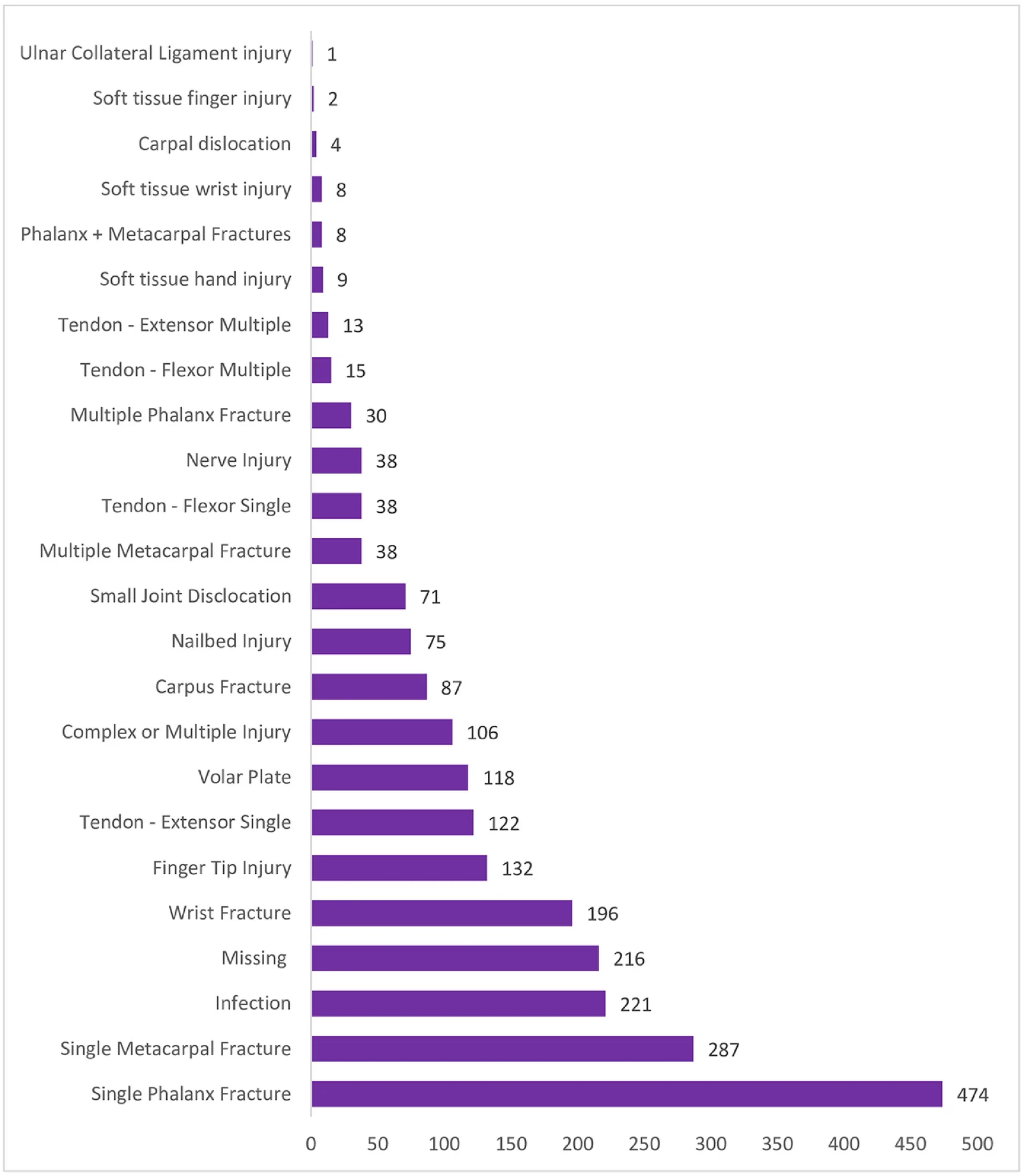
Injury classification.

**Fig. 2:**
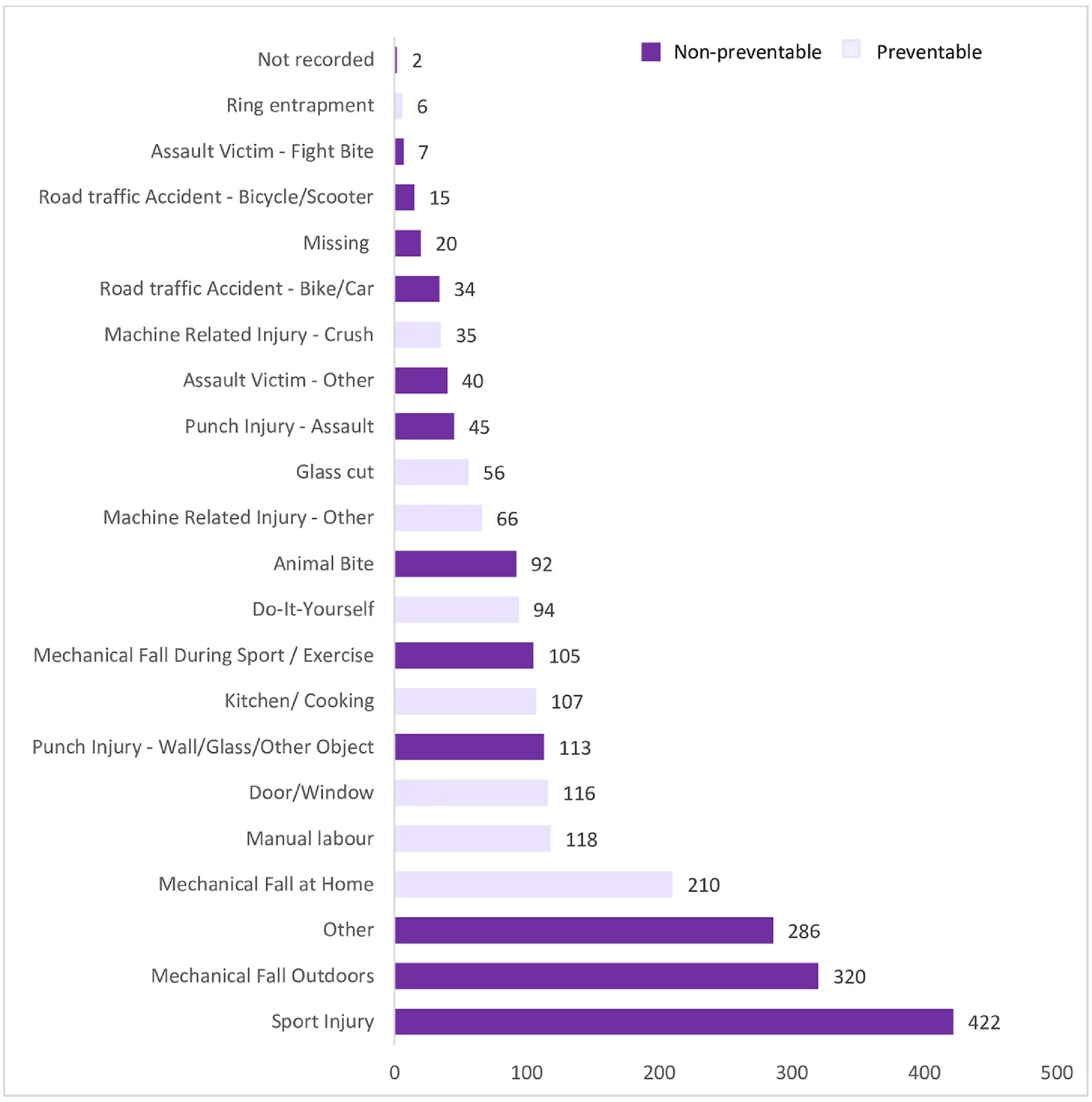
Mechanism of injury.

Regarding management, 27% of patients underwent a procedure either under general anaesthesia or local anaesthesia. The most common procedure was exploration under anaesthesia involving soft tissue debridement, repair and/or washout (Graph 3). The majority, 65%, were managed non-operatively. 43% of patients were followed up either in the clinic or hand therapy, 37% were discharged after assessment, 10% received care in the day surgery unit, and 4% required admission to the hospital.

**Fig. 3:**
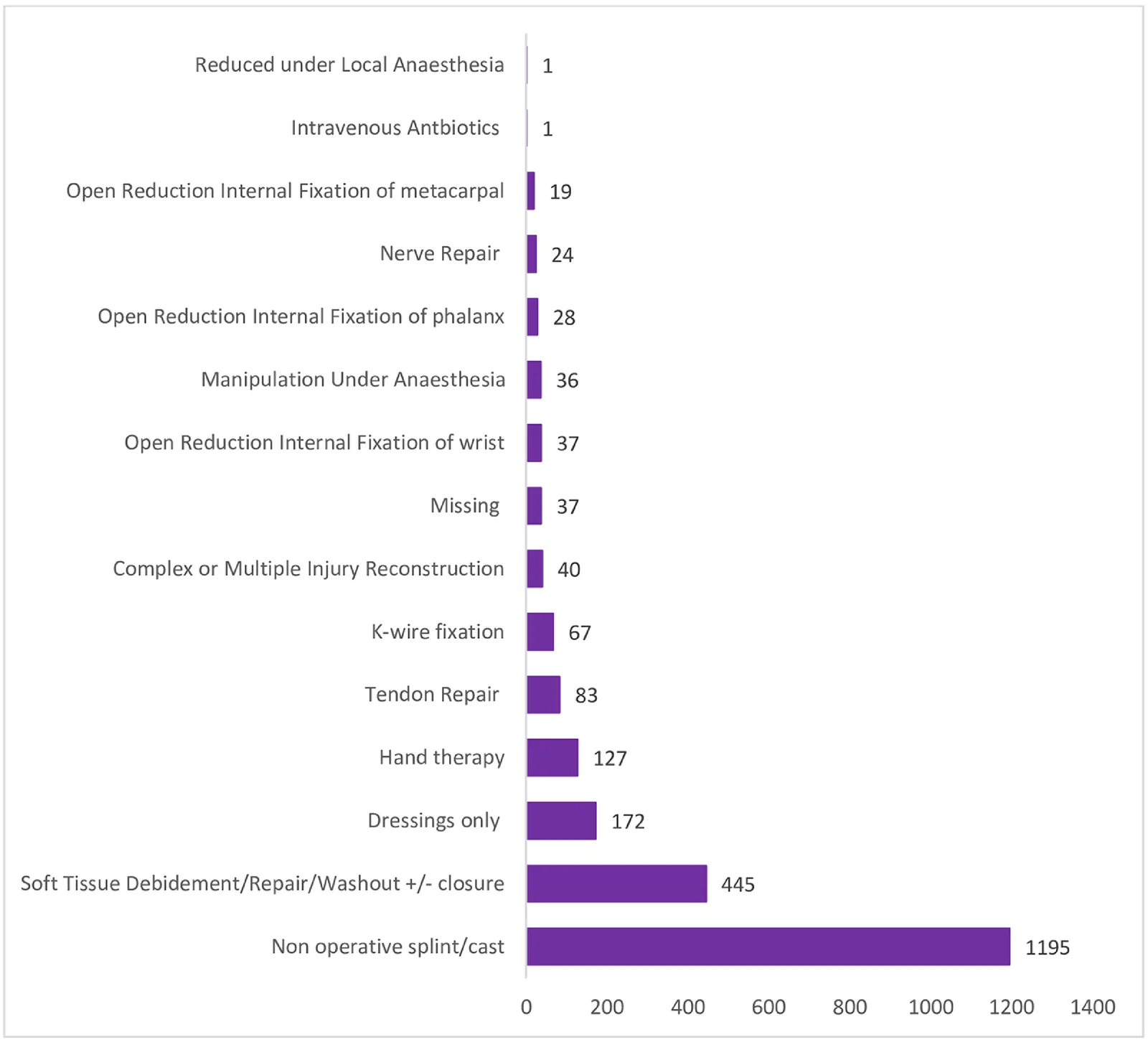
Management categories of the hand injuries.

There were significant differences between MTC and non-MTC groups across several domains. Time from injury to ED/GP presentation was shorter in MTC patients (median 0.0 vs 1.0 days, p<0.001), and time from ED/GP to specialist review was also reduced (median 1.0 vs 2.0 days, p<0.001) (as seen in Table 4, Table 5). The proportion of preventable injuries was slightly lower in MTCs compared to non-MTCs (40% vs 30%, p<0.001) (Table 6), although non-preventable injuries remained the majority in both groups. Operative management was more frequent in MTCs (45% vs 26%), while non-operative management predominated overall (p<0.001) (as observed in Table 7).

**Table 4:** Time from injury to first presentation (ED/GP) in MTC vs non-MTC patients.

	MTC N = 1,339^1^	Non-MTC N = 962^1^	p-value^2^
time from injury to ED/GP	0.0 (0.0, 1.0)	1.0 (0.0, 2.0)	<0.001
Unknown	17	16	

1 Median (Q1, Q3)

2 Wilcoxon rank sum test

**Table 5:** Time from ED/GP presentation to specialist review in MTC vs non-MTC patients.

	MTC N = 1,339^1^	Non-MTC N = 962^1^	p-value^2^
Time from ed/gp to spec	1.0 (0.0, 3.0)	2.0 (1.0, 6.0)	<0.001
Unknown	13	8	

1 Median (Q1, Q3)

2 Wilcoxon rank sum test

**Table 6:** Mechanism of injury (preventable vs non-preventable) in MTC vs non-MTC patients.

	MTC N = 1,339^1^	Non-MTC N = 962^1^	p-value^2^
MOI			<0.001
Non-preventable	808 (61%)	673 (70%)	
Preventable	524 (39%)	284 (30%)	
Unknown	7	5	

1 n (%)

2 Pearson’s Chi-squared test

**Table 7:** Management approach (operative vs non-operative) in MTC vs non-MTC patients.

	MTC N = 1,339^1^	Non-MTC N = 962^1^	p-value^2^
Management			<0.001
Non-operative	764 (57%)	691 (73%)	
Operative	568 (43%)	260 (27%)	
Unknown	6	12	

1 n (%)

2 Pearson’s Chi-squared test

The COVID and post-COVID cohorts were compared descriptively (Graphs 4,5,6,7,8,9), as the study was not powered to support formal statistical comparisons between cohorts. Overall, the cohorts were broadly similar in terms of patient numbers, median age, male predominance, and injury laterality. There were, however, some notable differences in injury mechanisms. In the COVID cohort, the most commonly recorded injuries in the previous cohort were wrist fractures, single phalangeal or metacarpal fractures, fingertip injuries, and infections. A similar distribution was observed in the current cohort, with single fractures of phalanges or metacarpals, infections, and wrist fractures remaining the most frequent injury types. Mechanisms of injury in the previous cohort were most commonly mechanical falls outdoors (16%) and mechanical falls at home (12%). The most common mechanisms were sports injuries (18.2%) and mechanical falls outdoors (13.9%). Most notably, in the current cohort, 35% were classified as preventable, compared to 50% in the previous cohort.

**Fig. 4:**
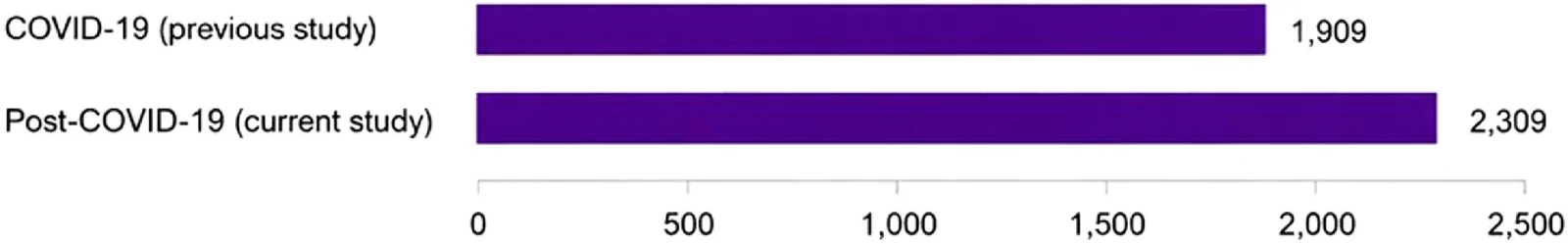
Cohort size of studies (n of patients).

**Fig. 5:**
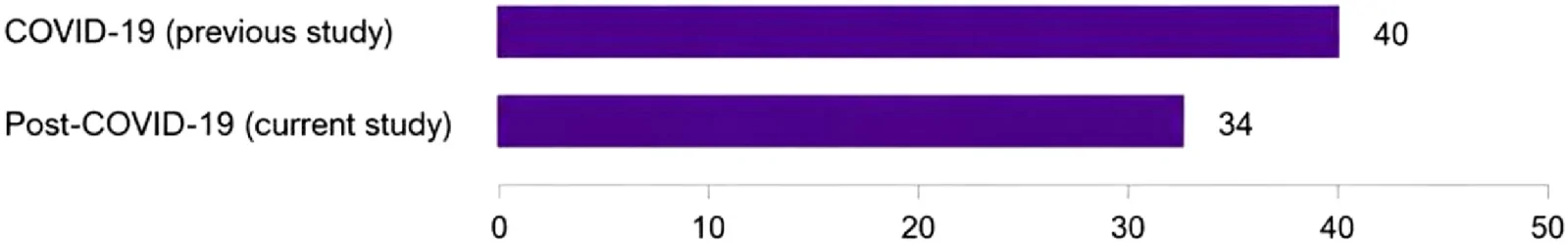
Median age (years) between studies.

**Fig. 6:**
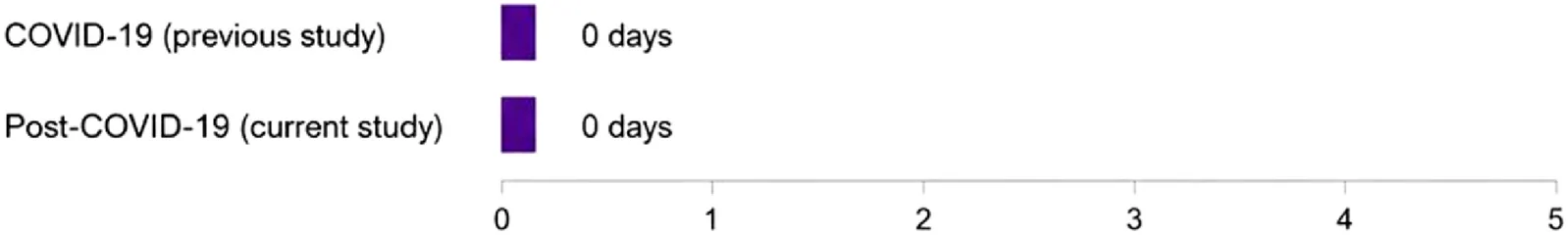
Median time from injury to presentation at ED or GP (days) between studies.

**Fig. 7:**
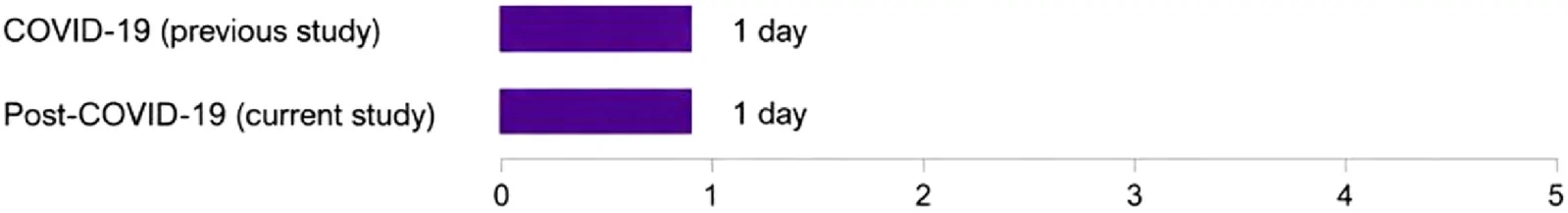
Median time from presentation to specialist review (days) between studies.

**Fig. 8:**
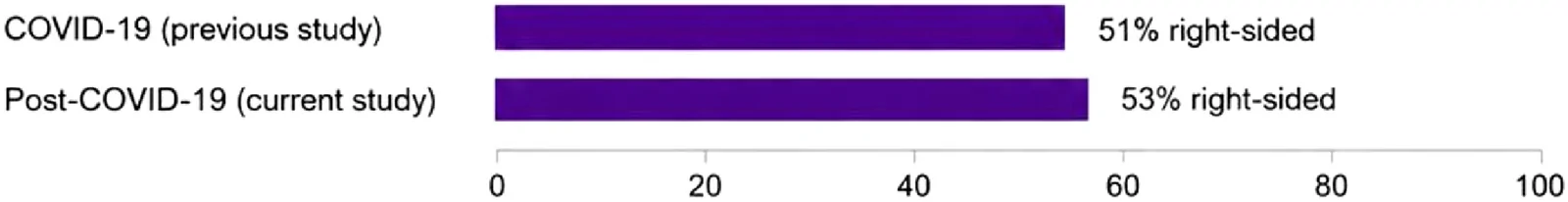
Laterality of injury (%) between studies.

**Fig. 9:**
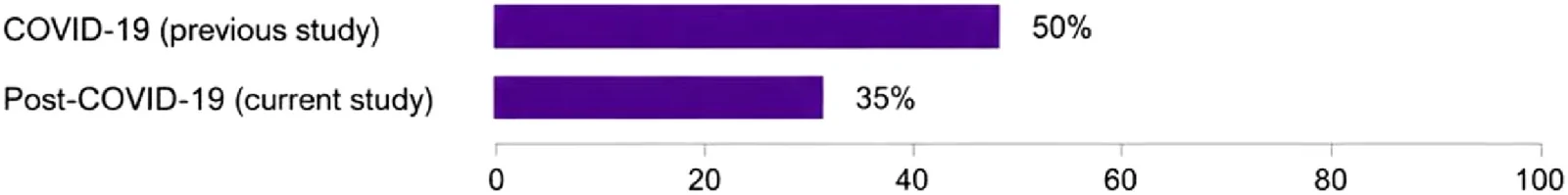
Preventability of injury (%) between studies.

## Discussion

This study revealed a substantial number of hand injuries recorded over the two-week data collection period. Compared to the COVID-19 lockdown data, there was a 21% increase in injuries, reflecting a moderate rise. This trend applies to both preventable and non-preventable injuries. A notable change was observed in sports-related injuries, which increased from 10.2% during the lockdown period to 22.7% in the post-COVID era, highlighting the impact of lifted restrictions on injury patterns, as more communal activities such as contact sports were allowed to resume. Perhaps the increase also takes into account increased participation after prolonged inactivity, but also expected deconditioning, leading to more injuries. Interestingly, the proportion of preventable injuries decreased from 50% to 35%, suggesting a shift in the overall distribution of injury types. This may likely account for the reduction in the median age of patients, from 40 to 34 years, as younger individuals are more likely to engage in sports, which saw a significant rise post-COVID. Interestingly, children under the age of 16 comprised only 14% of the cohort, suggesting that targeting this specific population may be of limited utility. Furthermore, sports injuries, which were the most common cause of injury among children, significantly influenced the proportion of preventable injuries in this subgroup. Only 18.62% of injuries in children were classified as preventable, compared to 35% in the overall cohort.

The observed differences between MTC and non-MTC pathways are consistent and clinically plausible, supporting the internal validity of the analysis. Shorter time intervals in MTCs may reflect direct conveyance pathways, higher case volumes, and more streamlined access to specialist input, whereas delays in non-MTC settings may be influenced by geographical factors. The similar distribution of preventable and non-preventable injuries suggests a comparable mix of cases, strengthening the reproducibility of these findings across settings. The higher operative rate in MTCs aligns with the centralisation of more complex cases. However, these systematic differences highlight the importance of accounting for care setting when interpreting outcomes, as differences in patient flow and service provision may otherwise confound comparisons.

The data from the two projects shared many similarities.^8^ Both observed that more men (60%) than women were involved in hand injuries, a trend consistent with findings in the broader literature. Other studies have noted that men tend to sustain more severe injuries, often involving wounds, whereas women are more likely to experience fractures, typically after a fall.^10^ Hand dominance demonstrated comparable patterns. In the sample, 52% of patients were right-handed (compared to 50% in the previous study), and 46% were left-handed (compared to 48%). Given the relatively balanced distribution, it would be of interest to explore whether there is any correlation between left-handedness and hand injuries. However, there is currently limited literature addressing this potential association.

One of the most common mechanisms of injury identified in both studies was manual labour.^11^ Manual labour injuries are often linked to several contributing factors, including inexperience or a lack of formal training as well as poor use of protective equipment, whether due to discomfort, inadequate training, or negligence. Additionally, research has shown that hand injuries predominantly affect young male labourers.^12^ This insight is valuable when considering prevention strategies. Potential measures could include worker education on safety practices, implementing safety mechanisms on workplace tools, and refining the design of instruments to minimise risk. This might involve optimising the size, shape, shock absorption, and overall ergonomics of tools.^13^ Regular safety inspections could further ensure compliance with these preventive measures, reducing unnecessary injuries in the workplace, while the use of gloves has also been studied before.^14^

DIY-related injuries dropped by half, from 8% to 4%, further reflecting the transition from lockdown behaviours to the post-pandemic era.^8^ Although trends like this one suggest that hand trauma has transitioned from predominantly domestic mechanisms during lockdown to more activity-based mechanisms in the post-pandemic period, door/window, kitchen and DIY injuries present quite frequently. Targeted education could focus on those high-risk activities. Hand injuries in the UK linked to power tools were most often traced to circular saws, which accounted for just over a quarter of the incidents, followed by hedge trimmers at roughly one-fifth, and electric drills at about one-sixth.^15^ It is also important to recognise the behavioural patterns that underpin many of these injuries, for instance, rushing to complete tasks, overconfidence when using familiar tools, and underestimating the force or motion of common household machinery. Complacency, distraction, and inadequate planning, particularly during routine tasks such as cooking or minor repairs, may also play an important role in sustaining these injuries, despite the seemingly low-risk environment. Instructional resources for safe tool use, ergonomic recommendations, and guidance on avoiding hand injuries in the kitchen could have a meaningful impact. This could also be in the form of a campaign involving workshops, speeches, theatrical animations for children etc. as reported before.^16^

### Limitations

The collection of specific mechanism-of-injury data allows for a more reliable characterisation of preventable injuries. However, there are limitations to the method used to define preventable versus non-preventable injuries. Rather than employing a free-text analysis on a case-by-case basis, the classification was based on predefined categories of injury mechanisms. This approach is pragmatic, aiming to encourage clinical collaborators across multiple sites to actively participate in data collection. By adopting this method, the study was able to achieve the most comprehensive data collection possible across all 24 sites and provided a replicable plan for descriptive analysis. Nonetheless, this approach has its drawbacks, particularly the potential loss of granularity in injury mechanisms, which could result in some degree of misclassification.

Many hand injuries are likely attributable to impatience or inexperience. While "carelessness" is challenging to document due to its subjective nature, it has been explored in other contexts. For instance, David et al. studied sugarcane crushers in India and their perception of hand injury risks during work.^17^ The study found a prevalent fatalistic attitude, which is difficult to overcome. The concept of “carelessness” is particularly subjective, challenging its utility in injury classification or prevention research. Nevertheless, safety education programmes must account for the socioeconomic and educational backgrounds of the target population, tailoring interventions to address these factors.

The two-week data collection period may be limiting, as it captures only a narrow snapshot of seasonal activities. For instance, the peak of sports activity typically occurs in spring, which would not be reflected in data collected during the winter. Similarly, outdoor falls are more likely in warmer seasons when people spend more time outside, potentially leading to the underrepresentation of these incidents in colder months. A more prolonged period of data collection would combat this. However, resources and compliance of collaborators would also need to be accounted for when determining this time period.

Likely due to the large number of data and involvement of multiple centres in the study, there is a considerable amount missing of data for a few categories. Notably, 9.4% of injury classifications were missing, while the status of preventability could not be assessed in 13.4% cases due to insufficient data. Despite the online version of the form, some fields were still not completed fully. Interestingly, while right-handedness was the most common (71.3%), the data had a large portion of missing data (20.6%). This missing data could hinder efforts to understand if left-handed individuals might have higher risks in certain tasks, perhaps due to tool design predominantly favouring right-handed users. Addressing this gap could improve understanding of how handedness affects injury patterns and prevention. The 13% of injuries marked as “Not Applicable” likely include injuries with unclear mechanisms. Addressing this category could involve refining classification protocols, which would lead to more actionable data and precise prevention strategies, in the context of the limitations of manually screening the large amount of data.

This study provides the first national dataset of hand injuries collected in a non-lockdown setting, offering a more accurate picture of typical injury patterns in the UK. The findings highlight key behavioural drivers, particularly increased sports participation, resumed manual labour, and common domestic activities, that contribute to the current burden of hand trauma. As such, this dataset serves as a benchmark for developing targeted prevention strategies in these high-risk areas and for evaluating future interventions aimed at reducing preventable hand injuries.

## Funding

There was no financial support from any organization for the submitted work.

## Declaration of competing interest

All authors have completed an ICJME conflict of interest form that can be provided if needed to the journal editorial team.
