# Supplementary material for: A national audit of hand injuries in UK
Source: JPRAS Open. 2026 Sep 28;52:582–91. doi: 10.1016/j.jpra.2026.09.022 (PMC13676084; doi:10.1016/j.jpra.2026.09.022)
Supplement: Supplementary file 1 [file mmc1.docx]

**JAPRAS Ref. No.: JPRAS-D-26-00879**

**Title: A National audit of hand injuries in UK**

**REVIEWER COMMENTS TABLE**

Dear Mr Reid,

Thank you for your review of this study and your work to date. We have addressed the reviewer’s comments in the revised manuscript and have uploaded both a clean and marked-up version for ease of review.

Please see below responses to the points raised by the reviewers.

We would also like to confirm that all the authors mentioned in the hand injury collaborative will be PubMed searchable in association with this publication, should it be published in your journal, including if changed to a short report?

| **Reviewer comment** | **Author Response** | **Revised Manuscript Line Number and Text Change** |
| --- | --- | --- |
| Editorial Feedback: |  |  |
| **LATEST FEEDBACK BEFORE TRANSFER TO OPEN ACCESS**:  Editorial Review: Overall it was felt that the manuscript was not compelling enough in its content for the main journal. It did not fulfill the requirements of a closed loop audit whereby change was implemented or standards were improved. In addition the robustness of characterization of preventable vs non preventable hand injuries was open to question, for example how are sports injuries non-preventable and a fall in the home preventable? this is subjective, and not broken down to a granular enough level to critique. Although the scale is laudable the robustness of the data set for the two weeks of capture may not be reflective of the rest of the year. In addition if the Manchester data was excluded because it was felt to be non representative, how can the authors claim/validate the rest of the data was representative. Finally the conclusions. around sports injuries doubling and DIY injuries halving etc are largely inferred , pre and post covid data comparative data of the  injury types have not been provided and hence this is not statistically robust. | We thank the reviewer for their comments regarding the overall impression of the manuscript and for suggesting an open-access journal. We are happy to proceed with this recommendation and have therefore submitted the manuscript to the suggested journal.  Regarding the fulfilment of closed-loop audit criteria, we accept the reviewer’s comment and have amended the title to remove the term “closed-loop audit”.  Regarding the characterisation of preventable injuries, including sports-related injuries, the relevant definitions and criteria are detailed in the “Sample Characteristics and Definition of Preventability” section. These were established a priori and applied consistently; therefore, no further changes have been made.  We apologise again for the exclusion of the Manchester data and acknowledge the considerable effort made by the centre in collecting these data. However, the volume provided was disproportionately low for an MTC. We acknowledge that we cannot claim that the remaining data are representative of all major trauma centres, but no such explicit claim was made in the manuscript.  Finally, we did not intend to make a direct comparison between pre-COVID and post-COVID periods. Our analysis primarily compares injury patterns during COVID-19 with those in the subsequent post-COVID period, as clarified in the Results and Discussion sections. | Title changed (removed ‘closed-loop’ terminology) |
| The manuscript presented reports pre covid, lockdown, and post covid data sets although the pre-covid and current cohorts were compared descriptively. It is not clear why this was and how these data sets can be best graphically demonstrated as this would make comparisons clearer and hence more impactful if displayed together. the manuscript also states manchester was excluded due to low numbers? this is unusual as the unit led the Hands First programme. Is this from a MTC perspective or a tertiary centre perspective? Would merit clarification. There does not seem to have been any ethics submitted for the study. Also how does the data set relate to the Hands First programme? | Thank you for your insightful comments.   - Thank you for this comment, we had decided to compare descriptively because there was not enough power to the data to perform an analysis. We think it is still quite important to keep the descriptive analoysis and provide a graph as seen in the edited version of the paper. The text has also been shortened as most data are reflected nicely on the graph. - Thank you for this important comment. We agree that Manchester, as a major trauma centre, would be expected to provide valuable data. However, the number of cases submitted during the study period was incredibly lower than anticipated. As we could not determine the reason for this under-recruitment, we considered the dataset unlikely to be representative of the centre's usual activity or case mix. To avoid introducing potential bias into the analyses, we elected to exclude these data. A new text has been added trying to explain the above in a better way. - As this was an audit ethical approval was not required. We have clarified this on the manuscript as seen below. Specifically, we now state that this project was classified as a clinical audit using the UK Health Research Authority (HRA) Decision Tool and therefore did not require Research Ethics Committee approval. - We thank the reviewer for this comment. The current audit is independent of the Hands First programme. We recognise the considerable contribution of the Hands First initiative in advancing hand trauma care through the development of standards, care pathways, and service improvements. The aim of the present study was different. Rather than evaluating service delivery or implementation of standards, this national audit aimed at characterising the epidemiology of hand injuries in the UK following the COVID-19 pandemic, with a particular focus on injury mechanisms and preventability. | - Graphs to compare COVID-19 vs post-COVID data added to edited version of manuscript submitted. The text has been slightly altered as seen on the document with tracked changes to not be repetitive - The text added in Methods section: ‘Unfortunately, the Manchester data set was excluded from the present study because the number of cases submitted was substantially lower than expected for an MTC and was therefore considered unlikely to be representative of the centre's usual case volume. To avoid introducing potential bias into the analyses, these data were excluded.’ - New text added: ‘**Ethical approval**   As this project was conducted as a national clinical audit, it was classified as an audit/service evaluation using the UK Health Research Authority (HRA) Decision Tool and did not require Research Ethics Committee approval. Local registration and approval were obtained at each participating centre in line with local governance requirements. |
| Reviewer #1 |  |  |
| Very short duration of Study.  Need to have at least a review of six months volume to arrive at meaningful conclusions. | Thank you for this comment. We agree that a longer data collection period would provide a more comprehensive representation of injury patterns and reduce the potential influence of seasonal variation. However, this study was designed in a way that the duration reflected the practical challenges of coordinating data collection across multiple UK centres while maintaining collaborator engagement, data quality and completeness. Despite the relatively short collection period, the study included over 2,000 patients, providing a substantial dataset for analysis. This limitation has already been acknowledged and discussed within the manuscript in the discussion session. | No changes indicated |
|  |  |  |
| This is a good paper to provide public, specifically in the UK, with a local epidemiological data. Although it does not generate any direct clue of information about baseline for prevention measure, this paper would still be good as comparative data. The authors use the previous study during lockdown due to COVID-19 to compare to the current study.  There are few concerns for the authors to respond:  1. The term "prevention" in the title of the manuscript does not directly reflect the content of the study. Thus, the term "prevention" is misleading.  2. The title of the Tables could be revised with a proper numbering order. while avoiding subdivision. | Thank you for your kind comment. To address the specific points:   1. The title should be revised as seen below indeed. 2. Table titles and numbers revised as seen below. | New title: A National closed-loop audit of hand injuries in UK  New table titles and numbering:  Table 1: Number of hand injuries in Major Trauma Centres (MTC)  Table 2: Number of hand injuries in Non-Major Trauma Centres (Non-MTC)  Table 3: Missing data/Unknown Hospitals  Table 4: Time from injury to first presentation (ED/GP) in MTC vs non-MTC patients  Table 5: Time from ED/GP presentation to specialist review in MTC vs non-MTC patients  Table 6: Mechanism of injury (preventable vs non-preventable) in MTC vs non-MTC patients  Table 7: Management approach (operative vs non-operative) in MTC vs non-MTC patients |
| Editorial Office: |  |  |
| Please include statements for Conflict of Interest, Funding and Ethical Approval at the end of your text just before the references, please refer to the Guide for Authors for further information. | Thank you for this, a statement has been added just before the references as seen below: | Text added:  ‘Conflict of interest statement:  All authors have completed an ICJME conflict of interest form that can be provided if needed to the journal editorial team.  Funding  There was no financial support from any organization for the submitted work.  Ethical Approval  The study was approved at each contributing site with local research governance approval prior to data collection. This work was considered audit following the HRA decision making process.’ |
