# Supplementary material for: A national audit of hand injuries in UK
Source: JPRAS Open. 2026 Sep 28;52:582–91. doi: 10.1016/j.jpra.2026.09.022 (PMC13676084; doi:10.1016/j.jpra.2026.09.022)
Supplement: Supplementary file 2 [file mmc2.docx]

**Lay summary**

A UK-wide study of hand injuries post-COVID shows over one-third are preventable, often linked to everyday activities like manual work and DIY. Targeted safety education and awareness could reduce injury rates and healthcare burden.
